# 32. Rheumatoid pachymeningitis: a rare extra-articular manifestation of rheumatoid arthritis

**DOI:** 10.1093/rap/rky033.023

**Published:** 2018-09-20

**Authors:** Alice Mason, Christopher Chan, George Pengas, Christopher Holroyd

**Affiliations:** 1 Rheumatology, University Hospital Southampton, Southampton, UNITED KINGDOM; 2 Neurology, University Hospital Southampton, Southampton, UNITED KINGDOM

**Introduction:** We present the case of a 72 year old lady with longstanding, poorly controlled sero-positive, erosive rheumatoid arthritis (RA) presenting with multiple recurrent episodes of left sided weakness. Magnetic resonance imaging (MRI) of the brain demonstrated pachymeningitis and these episodes were presumed to be focal seizures secondary to meningeal irritation. Infection, malignancy and other autoimmune disease were excluded as a cause and the patient did not improve clinically with several courses of antibiotics. Dural biopsy was inconclusive. She was treated as presumed rheumatoid pachymeninigitis with high dose steroid and has now been discharged with follow up clinic and MRI scanning scheduled. This case demonstrates the difficulty of making this diagnosis of rheumatoid pachymeninigitis - a rare extra-articular manifestation of RA which has a poor prognosis. A literature review suggests that patients affected tend to be rheumatoid factor positive, have poorly controlled and a long history of RA. Peak incidence is between 50 and 80 years-old. Focal seizure is a common presentation. We discuss this unusual case in detail and review the literature, pathophysiology, presentation, investigation and treatment of this condition.

**Case description:** A 72 year old lady with sero-positive (anti-CCP antibody> 340, rheumatoid factor negative) erosive RA was admitted under the stroke team with a three-day history of fluctuating right arm and leg weakness whilst on a cruise. Transient ischaemic attack was suspected. Initial CT brain did not show any abnormality. The episodes continued and an MRI brain was requested and showed a collection in the left parafalcine region and leptomeningeal enhancement overlying the medial aspect of the left frontal and parietal lobes at the vertex, which extended over the left posterior aspect of the parietal and posterosuperior occipital lobes. The patient was started on levetiracetam for focal seizures secondary to meningeal irritation and antibiotics were started for a presumed infection. Methotrexate was held. White blood cell count was 8.1 with neutrophils of 5.8 and lympohcytes of 0.8. LP was done and her CSF findings were unremarkable: white blood cells <1, protein 594, IgG - 0.04, gram-stain and cultures, including tuberculosis (TB) culture were negative. The result did not reflect infection and thus antibiotic was stopped. Evidence of TB, sarcoidosis, lymphoma and other infective and inflammatory causes were looked for. Angiotensin converting enzyme, lactate dehydrogenase, immunoglobulins (including IgG4) and electrophoresis were all normal. ANA and ANCA was negative. Cryptococcal antigen, quantiferon TB gold test, syphilis serology and 16S polymerase chain reaction were negative. CT chest, abdomen and pelvis showed no evidence of a lymphoma or other malignancy. There were no features to suggest a systemic inflammatory granulomatous process. The diagnosis of rheumatoid associated pachymeningitis was considered. She had a diagnosis of rheumatoid arthritis made over 40 years ago. She took methotrexate 15 mg once weekly, sulfasalazine 1 G twice daily, and prednisolone 7.5 mg once daily for this. Her arthritis had never achieved disease remission, with moderate disease activity present at most consultations. Her c-reactive protein was persistently mildly raised between 10 and 30 mg/L with an erythrocyte sedimentation rate of 30-50 mm/hr. At the time of admission there was no evidence of active synovitis. As the cause was in question, dural biopsy was performed; biopsy of the dura and pia mater showed basophilic material containing scattered neutrophils and apoptotic debris; the appearances of which suggests inflammatory debris. No neoplastic cells were seen. Two gram positive organisms were seen in the biopsy, this was suspected to be a contaminant, but was treated with antibiotics as a precaution. She was given two weeks of chloramphenicol followed by ten days of meropenem. With no clinical improvement, MRI brain was repeated. This was difficult to interpret in view of the recent biopsy, but did not show any definite improvement. A diagnosis of presumed rheumatoid associated pachymeningitis was made. She was treated with three days of intravenous methylprednisolone (1 gram per day), which was then switched to oral prednisolone 60 mg daily. Treatment with methotrexate was restarted. The patient has now been discharged on a reducing regime of prednisolone with a plan to repeat her MRI in two months. Seizure frequency has lessoned and we await to see if she continues to show further improvement.

**Discussion:** This patient presented with typical clinical and radiologic features of rheumatoid pachymeningitis. As dural biopsy was inconclusive, several differential diagnoses were ruled out. This rare extra-articular manifestation of RA, which can be caused by both fibrinoid deposits and rheumatoid nodules, is often a diagnosis made on biopsy or by exclusion. Patients frequently have a long history of poorly controlled disease and positive serology. MRI imaging reveals areas of thickened meninges, seen as areas of focal hyperintensity and enhancement. Other diagnoses must be excluded, including malignancy (most commonly lymphoma), infection and other autoimmune disease such as sarcoidosis. Diagnosis has been difficult in this case as dural biopsy was inconclusive, but other diagnoses have been excluded. The presentation is rare and there is little in the literature regarding how to monitor and treat these patients. Prognosis is generally poor with reports of death within six months for many patients.

**Key Learning Points:** Rheumatoid pachymeninigitis is a rare extra-articular manifestation of rheumatoid arthritis. It commonly presents with focal seizures in patients with a long history of poorly controlled disease and positive serology. Diagnosis is frequently made by exclusion. Several other differential diagnoses need to be ruled out: malignancy, infection, other autoimmune disease. No guidelines are available on treatment. Previous cases have been treated with steroids, cyclophosphamide and rituximab.

**Disclosure: A. Mason:** None. **C. Chan:** None. **G. Pengas:** None. **C. Holroyd:** None.

